# Inhibition of Osteoclast Activation by Phloretin through Disturbing ***α***v***β***3 Integrin-c-Src Pathway

**DOI:** 10.1155/2015/680145

**Published:** 2015-03-05

**Authors:** Eun-Jung Lee, Jung-Lye Kim, Ju-Hyun Gong, Sin-Hye Park, Young-Hee Kang

**Affiliations:** Department of Food Science and Nutrition, Hallym University, Chuncheon 200-702, Republic of Korea

## Abstract

This study was to explore the sequential signaling of disorganization of the actin cytoskeletal architecture by phloretin. RAW 264.7 macrophages were incubated with 1–20 *μ*M phloretin for 5 days in the presence of RANKL. C57BL/6 mice were ovariectomized (OVX) and orally treated with 10 mg/kg phloretin once a day for 8 weeks. Phloretin allayed RANKL stimulated formation of actin podosomes with the concomitant retardation of the vinculin activation. Oral administration of phloretin suppressed the induction of femoral gelsolin and vinculin in OVX mice. The RANK-RANKL interaction resulted in the *α*v*β*3 integrin induction, which was demoted by phloretin. The RANKL induction of actin rings and vacuolar-type H^+^-ATPase entailed Pyk2 phosphorylation and c-Src and c-Cbl induction, all of which were blunted by phloretin. Similar inhibition was also observed in phloretin-exposed OVX mouse femoral bone tissues with decreased trabecular collagen formation. Phloretin suppressed the paxillin induction in RANKL-activated osteoclasts and in OVX epiphyseal bone tissues. Also, phloretin attenuated the Syk phosphorylation and phospholipase C*γ* induction by RANKL in osteoclasts. These results suggest that phloretin was an inhibitor of actin podosomes and sealing zone, disrupting *α*v*β*3 integrin-c-Src-Pyk2/Syk signaling pathway for the regulation of actin cytoskeletal organization in osteoclasts.

## 1. Introduction

Large multinucleated osteoclasts specialized for bone resorption play a key role in bone turnover [1, 2]. A relatively excessive activity of bone-resorptive osteoclasts often eventuates in the common bone disorder osteoporosis [3]. The magnitude of bone resorption of osteoclasts reflects their prevailing number and matrix-degrading capacity [4]. Osteoclast differentiation is regulated by macrophage colony-stimulating factor and receptor activator of nuclear factor *κ*B ligand (RANKL) interacting with their respective receptors of c-fms and RANK, on monocyte/macrophage precursors* in vitro* and* in vivo* [2, 5]. Accordingly, understanding the means by which osteoclasts resorb bone conveys both physiological significance and clinical significance. Estrogen deficiency after menopause frequently accelerates osteoclastic bone resorption, leading to postmenopausal osteoporosis characterized by low bone mass and microarchitectural deterioration of bone tissues [6]. First-line pharmacological therapies for postmenopausal osteoporosis include agents inhibiting bone resorption, thereby improving bone strength and reducing osteoporotic fracture [7–9].

The resorptive capacity of mature osteoclasts is largely influenced by cytoskeletal reorganization [10, 11]. Upon contact with bone matrix, osteoclasts demarcate an acidified resorption compartment between the apical membrane and the bone surface by actin cytoskeleton reorganization into a podosome belt that forms a gasket to restrict lacunar acid leakage [12, 13]. The ability of osteoclasts to optimally resorb bone depends upon the formation of actin rings or sealing zones [12, 14]. RANKL organizes the cytoskeleton of mature resorptive cells, thereby stimulating their capacity to degrade bone [5]. To suitably rearrange the osteoclast cytoskeleton by RANKL entails the induction of RANK association with c-Src, which links RANK to the *α*v*β*3 integrin [15, 16]. c-Src permits the receptor/kinase complex to organize the osteoclast cytoskeleton by activating the cytoskeleton-organizing molecules [13, 14]. Thus, potential agents targeting the osteoclast formation of actin rings or sealing zones may display favorable effects in combating resorptive bone diseases.

Complementary therapies to alleviate osteoclast bone resorption and postmenopausal osteoporosis include use of phytoestrogens such as isoflavones and prenylated flavonoids as a hormone therapy alternative [17, 18]. Phytoestrogens have estrogen-like biological activity primarily via binding to estrogen receptors, which is effective in preventing hot flashes and menopausal bone loss [19]. However, the efficacy of phytoestrogens is still debatable [20]. There is much evidence toward lack of effectiveness of isoflavones derived from soy or red clover, even in large doses [21]. On the other hand, other polyphenols have been shown to prevent bone loss through blocking osteoclast formation and bone resorption [22, 23]. Our previous study revealed that the polyphenol phloretin inhibited RANKL-induced osteoclast formation and bone resorption [24].

**Figure 1:**
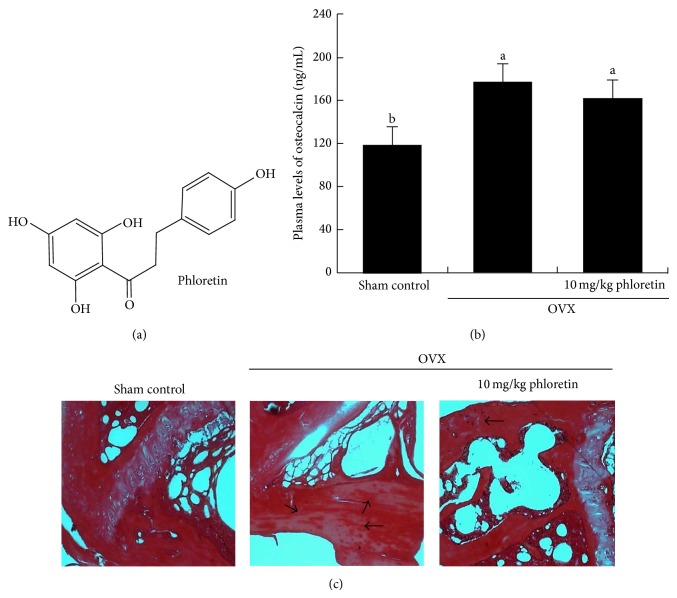
Chemical structure of phloretin (a), plasma osteocalcin level (b), and induction of trabecular collagen formation (c) in ovariectomized (OVX) mouse femoral bone tissues. Plasma osteocalcin level was measured by using an ELISA kit in OVX mice orally administrated with 10 mg/kg phloretin daily for 8 weeks. Plasma osteocalcin levels (mean ± SEM, *n* = 3) in bar graphs not sharing a letter indicate significant difference at *P* < 0.05. Picrosirius red staining was conducted for detecting trabecular bone collagen. The black arrows indicate the depletion of collagen in trabecular bones of the epiphysis. Representative images were visualized under light microscopy. Magnification: 40-fold.

Based on the evidence that plant compounds such as green tea (−)-epigallocatechin gallate (EGCG) may block pathological bone loss and optimize bone health [23], this study investigated that phloretin (Figure 1(a)) antagonizing bone resorption allayed actin cytoskeletal reorganization into podosome belts in osteoclasts. This study further elucidated the sequential molecular events and signaling for the disorganization of actin cytoskeletal architecture induced by phloretin. The osteoprotective mechanisms of phloretin were also examined in femoral bone tissues of ovariectomized (OVX) mice.

## 2. Materials and Methods

### 2.1. Materials

Fetal bovine serum (FBS), penicillin-streptomycin, and trypsin-EDTA were purchased from Lonza (Walkersville, MD). Minimum Essential Medium Alpha Medium (*α*-MEM), Dulbecco's modified eagle's media (DMEM), and phloretin were purchased from Sigma-Aldrich Chemicals (St. Louis, MO), as were all other reagents, unless specifically stated elsewhere. RANKL was obtained from PeproTech (Rocky Hill, NJ). Anti-mouse gelsolin, anti-mouse vinculin, anti-mouse Syk, anti-mouse phospho-Syk, and anti-mouse phospho-paxillin were purchased from Abcam (Cambridge, UK). Anti-rabbit phospho-vinculin was obtained from Biorbyt (Cambridge, UK). Antibodies of mouse integrin *α*v, mouse phospho-Pyk2, and mouse vacuolar-type H(+)-ATPase (V-ATPase) were obtained from Santa Cruz Biotechnology (Santa Cruz, CA). Antibodies of mouse integrin *β*3, mouse phospho-PLC*γ*, mouse Akt, mouse phospho-Akt, and mouse c-Cbl were purchased from Cell Signaling Technology (Beverly, MA). Mouse c-Src antibody was provided by Millipore (Billerica, MA) and LY294002 obtained by Calbiochem (Billerica, MA). Rhodamine phalloidin was obtained from Life Technologies (Carlsbad, CA). Horseradish peroxidase-conjugated goat anti-rabbit IgG was provided by Jackson ImmunoResearch Laboratories (West Grove, PA).

### 2.2. Osteoclast Differentiation and Activation of Raw 264.7 Cells

Murine macrophage Raw 264.7 cells (American Type Culture Collection, Manassas, VA) were cultured in DMEM containing 10% FBS, 2 mM glutamine, 100 U/mL penicillin, and 100 *μ*g/mL streptomycin at 37°C in a humidified atmosphere of 5% CO_2_ in air. For the osteoclast differentiation, Raw 264.7 cells were plated on well plates at the density of 1 × 10^4^ cells/mL and cultured for 5 days in *α*-MEM containing 10% FBS and 50 ng/mL RANKL in the absence and presence of 1–20 *μ*M phloretin. Cell culture medium was newly changed every 2 days. Phloretin was dissolved in dimethyl sulfoxide (DMSO) for live culture with cells; a final culture concentration of DMSO was <0.5%.

### 2.3. Animals and Ovariectomy

To investigate mechanistic effects of phloretin on antiosteoporotic activity in an estrogen-deficient animal model, this study introduced techniques of ovariectomy for mimicking estrogen deprivation or senescent menopause [24]. C57BL/6 mice (11 weeks of age, 20–25 g) were provided by the Experimental Animal Center, Hallym University, and kept on a 12-hour light/dark cycle at 20–25°C with 60% relative humidity under specific pathogen-free conditions. Mice were fed a nonpurified diet (RodFeedTM, DBL, Umsung, Korea) during the 8-week experimental period with free access to water ad libitum at the animal facility of Hallym University. The animals were allowed to acclimatize for a week before beginning the experiments. For the ovariectomy surgery, 11-week-old female animals were anesthetized using a ketamine/rompun cocktail (40 mg ketamine and 10 mg rompun/kg body weight) for either a sham operation (Sham control) or bilateral oophorectomy (ovariectomy, OVX). All efforts for surgery were made to minimize suffering. Mice receiving surgical OVX were orally treated with 10 mg/kg phloretin (dissolved in 20 *μ*L ethanol) once a day for 8 weeks (9 mice of each group). OVX-alone mice orally received phloretin vehicle. After 8 weeks following ovariectomy surgery, the final body weight and average daily gain highly increased in OVX mice, as compared to sham-operated control mice. The enhanced average daily gain in OVX mice was significantly diminished by administrating phloretin. No significant difference was observed in the average daily feed intake of each mouse group. A marked reduction of the uterus in size and wet weight due to OVX was observed, whereas the oral administration of phloretin to OVX mice alleviated the atrophy, being concomitant with increased wet weight of OVX mouse uterus [25].

Sera obtained from blood samples by centrifugation (3,000 rpm, 10 min) were stored at −70°C prior to analyses. Plasma osteocalcin levels were measured by using a commercial enzyme-linked immunosorbent assay (ELISA) kit (USCN Life Science, Wuhan, China), according to the manufacturer's instruction. All animal experiments were performed in* strict* accordance with the Hallym University's Guidelines for the Care and Use of Laboratory Animals. The protocol was approved by the Committee on the Ethics of Animal Experiments of the Hallym University (permit number: Hallym 2011-24).

### 2.4. Collagen Staining

Femoral bone tissues were decalcified in decalcifying solution (Sigma-Aldrich Chemicals) and dehydrated in a graded series of ethanol solutions for 18 h. For the histological staining of picrosirius red, femoral bone tissues were then embedded in paraffin and cut into 7 *μ*m sections in thickness. Picrosirius red staining was commercially used for detecting bone collagen. The sections were placed on glass slides, deparaffinated, and hydrated with xylene and graded alcohol. These sections were incubated with picrosirius red (Sigma-Aldrich Chemicals) solution overnight at RT. After each slide was mounted in VectaMount Mounting Medium (Vector Laboratories, Burlingame, CA), images were taken using an Axiomager optical microscope system (Zeiss, Oberkochen, Germany).

### 2.5. Actin Ring Staining

Raw 264.7 cells on 24-well plates were differentiated for 5 days in *α*-MEM containing 10% FBS and 50 ng/mL RANKL in the absence and presence of 1–20 *μ*M phloretin. Cells were fixed in 4% formaldehyde for 10 min and washed with prewarmed phosphate-buffered saline (PBS). Subsequently, 10 units of the fluorescent dye rhodamine phalloidin was added to cells and incubated for 20 min. Nuclear staining was also conducted by using 4 mg/mL 4′,6-diamidino-2-phenylindole (DAPI). Fluorescent images were taken with an Axiomager Optical fluorescence microscope.

### 2.6. Western Blot Analysis

Western blot analysis was conducted with cell lysates prepared from Raw 264.7 cells differentiated with RANKL. Equal amounts of lysate proteins were electrophoresed on 6–15% SDS-PAGE gels and transferred onto a nitrocellulose membrane. Nonspecific binding was blocked by soaking membranes in a TBS-T buffer (50 mM Tris-HCl (pH 7.5), 150 mM NaCl, and 0.1% Tween 20) containing 3% bovine serum albumin or 5% nonfat milk for 3 h. The membranes were incubated with antigelsolin, antivinculin, anti-phospho-vinculin anti-mouse integrin *α*v, anti-mouse integrin *β*3, anti-mouse c-Src, anti-phospho-Pyk2, anti-mouse phospho-Akt, anti-mouse PLC*γ*, and mouse c-Cbl as a primary antibody. The membranes were then incubated with goat anti-rabbit IgG conjugated with horseradish peroxidase as a secondary antibody. The protein levels on gels were measured by using SuperSignal West Pico Chemiluminescence detection reagents (Pierce Biotechnology, Rockford, IL) and Konica X-ray film (Konica, Tokyo, Japan). Incubation with anti-*β*-actin was conducted for comparative control.

The bone fragments were trimmed free of soft tissue and washed to remove contaminants with PBS and incubated at 4°C overnight in 1.2 M HCl to demineralize the bone tissue. The demineralized bone tissue was water-washed and incubated for 72 h at 4°C in 100 mM Tris lysis buffer (pH 7.4) containing 6 M guanidine-HCl and protease inhibitors cocktail. The supernatant (crude extract proteins) was collected by centrifugation. The residue was further extracted by repeating the above extraction procedure and the collected supernatant was combined with the first supernatant. Western blot analysis was carried out with extract proteins prepared from femoral bone tissues.

### 2.7. Immunochemical Staining

Raw 264.7 cells grown on glass coverslips were differentiated for 5 days in *α*-MEM containing 10% FBS and 50 ng/mL RANKL in the absence and presence of 1–20 *μ*M phloretin and fixed with 4% ice-cold formaldehyde. To make cells permeable, 0.1% Triton X-100 was treated to cells. After blocking nonspecific binding with 20% FBS in PBS for 1 h at room temperature, the primary antibodies of V-ATPase and phospho-paxillin were added and incubated overnight at 4°C. After several washes with PBS-Tween 20, cells were incubated with anti-rabbit IgG conjugated with a fluorescent dye fluorescein isothiocyanate (FITC). Subsequently, following wash cells were incubated at 4°C for 20 min with phalloidin and counterstained with DAPI for 15 min at room temperature. The cells were mounted on the slide and observed by a fluorescence microscopy (Carl Zeiss).

Femoral bone tissues were decalcified and dehydrated in a graded series of ethanol solutions. Femoral bone tissues were then embedded in paraffin and cut into 3–5 *μ*m sections in thickness. For the immunohistochemical staining of Pyk2 and paxillin, the sections were placed on glass slides, deparaffinated, and hydrated with xylene and graded alcohol. The tissue sections were then subject to incubation with 0.5% H_2_O_2_ in methanol for the removal of endogenous peroxidase. The nonspecific antibody binding site was blocked by using 3% BSA in PBS-T for 1 h, followed by 1 h incubation with goat anti-mouse Pyk2 and paxillin. The tissue sections were incubated for 1 h with peroxidase-conjugated anti-goat IgG. The integuments were developed with 3,3′-diaminobenzidine as a substrate for 1 min and counterstained with methyl green for 10 min and with eosin for 1 min at RT. After each slide was mounted in VectaMount Mounting Medium, images were taken using an Axiomager optical microscope system. The TRAP activity was measured by the image analysis program of the microscope system.

### 2.8. Statistical Analyses

The results were expressed as mean ± SEM for each treatment group in each experiment. Statistical analyses were performed using Statistical Analysis Systems statistical software package (SAS Institute, Cary, NC). Significance was determined by one-way analysis of variance, followed by Duncan range test for multiple comparisons. Differences were considered significant at *P* < 0.05.

## 3. Results

### 3.1. Inhibitory Effect of Phloretin on Osteocalcin Production and Femoral Collagen Synthesis

Long-term reduction in bone osteocalcin levels may induce the formation of immature bone and that osteocalcin is one of the factors affecting bone turnover [26]. In our previous study, OVX reduced bone mineral density of mouse femurs, reduced serum 17*β*-estradiol level, and enhanced serum RANKL/osteoprotegerin ratio with uterine atrophy [25]. In the current study plasma osteocalcin level in mice increased after 8-week surgical OVX, compared to sham-operated mice (Figure 1(b)). In OVX mice receiving 10 mg/kg phloretin its plasma level had a declining tendency.

Picrosirius red staining shows the arrangements of collagen fibers in extracellular matrix of bones. In femoral trabecular bone tissues of OVX mice the intensity of dark-red stain was diminished, compared to sham-operated mice (Figure 1(c)). There was whitish patchy marks (black arrows) observed in femoral bone tissues of OVX mice. In marked contrast, picrosirius red dye staining of trabecular bone tissues of OVX mice treated with phloretin was indistinguishable from that of sham control animals (Figure 1(c)). Accordingly, phloretin may restore collagen formation plummeted after surgical OVX.

### 3.2. Inhibition of Actin Ring Formation and V-ATPase Production by Phloretin

**Figure 2:**
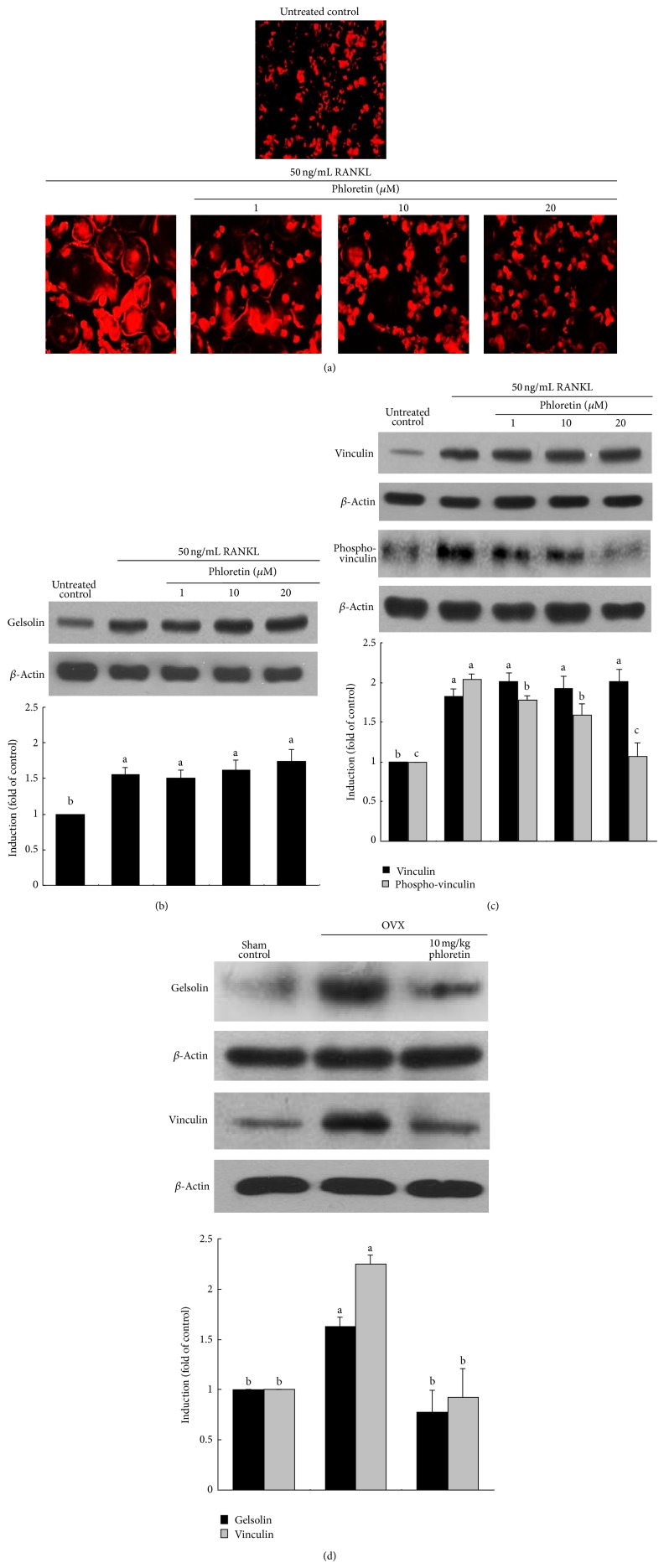
Inhibitory effects of phloretin on RANKL-induced actin ring formation (a), and induction and phosphorylation of gelsolin and vinculin (b, c, and d). Raw 264.7 macrophages were cultured in *α*-MEM for 5 days in the absence and presence of 1–20 *μ*M phloretin. Ovariectomized (OVX) mice were orally treated with 10 mg/kg/day phloretin daily for 8 weeks. RANKL-differentiated cells were fixed in 4% paraformaldehyde for 10 min and rhodamine phalloidin was added to fixed cells (a). Fluorescent images were taken with a fluorescence microscope. Original magnification of microscopic images (*n* = 3), 400-fold. Cell lysates and bone tissue extracts were subject to SDS-PAGE and Western blot analysis with a primary antibody against gelsolin, vinculin, or phospho-vinculin (b, c, and d). Representative blot data were obtained from three independent experiments, and *β*-actin protein was used as an internal control. The bar graphs (mean ± SEM) in the bottom panels represent quantitative results of blots obtained from a densitometer. Respective values not sharing a common letter are different at *P* < 0.05.

Actin ring is a unique cytoskeletal structure for the formation of the actin-rich sealing zone responsible for optimal osteoclastic activity and bone resorption [13]. The formation of actin rings was elevated in RANKL-treated osteoclasts, evidenced by the phalloidin staining (Figure 2(a)). When 1–20 *μ*M phloretin was treated to those osteoclasts, the formation of actin rings was noticeably retarded by administrating ≥10 *μ*M phloretin. Accordingly, phloretin was effective in retarding the RANKL-responsive osteoclast activation and cytoskeletal reorganization. RANKL highly induced cellular expression of gelsolin and vinculin (Figure 2(b)). However, phloretin did not influence the RANKL induction of these actin-binding proteins. Nevertheless, this compound inhibited the RANKL activation of vinculin in a dose-dependent manner (Figure 2(c)). On the other hand, the administration of phloretin to OVX mice suppressed the induction of gelsolin and vinculin in femoral bone tissues (Figure 2(d)).

**Figure 3:**
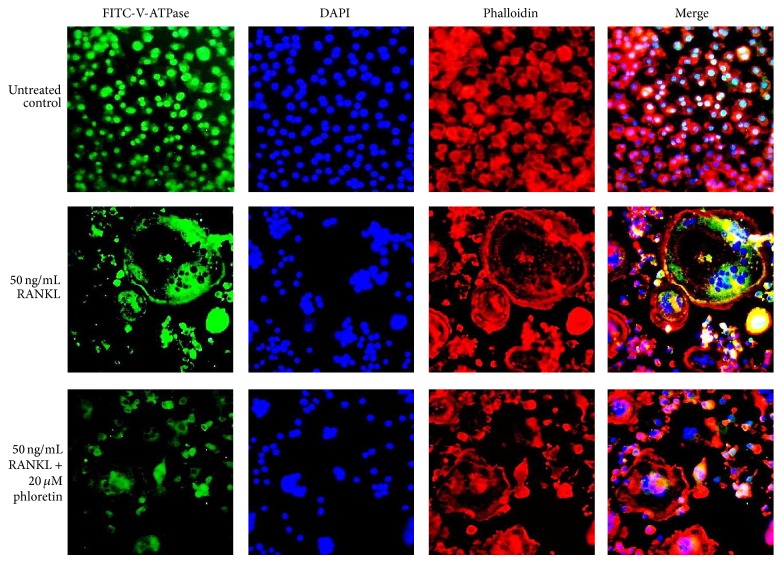
Suppressive effects of phloretin on RANKL-induced vacuolar-type H(+)-ATPase (V-ATPase) expression and actin ring formation. Raw 264.7 macrophages were cultured in *α*-MEM for 5 days in the absence and presence of 1–20 *μ*M phloretin. RANKL-differentiated cells were fixed in 4% paraformaldehyde for 10 min. Immunocytochemical analysis was conducted with the primary V-ATPase antibody and anti-rabbit IgG conjugated with FITC. Subsequently, cells were incubated with phalloidin and counterstained with DAPI. Fluorescent images were taken with a fluorescence microscope. Original magnification of microscopic images (*n* = 3), 400-fold.

The V-ATPase proton pump is present in the ruffled border plasma membrane of bone-resorbing osteoclasts, mediating extracellular acidification for bone demineralization during bone resorption [27]. In the RANKL-activated osteoclasts the V-ATPase (green) was distributed in the cytoplasm around the nucleus and with frequent colocalization in the area circumscribed by the peripheral ring belt of F-actin (Figure 3). However, osteoclasts present in the phloretin milieu lacked this dense ring-like pattern, and the V-ATPase protein was relatively depleted at the cell periphery (Figure 3).

### 3.3. Blockade of RANKL-Induced Integrin *α*v*β*3-cSrc-Pyk2 Signaling by Phloretin

**Figure 4:**
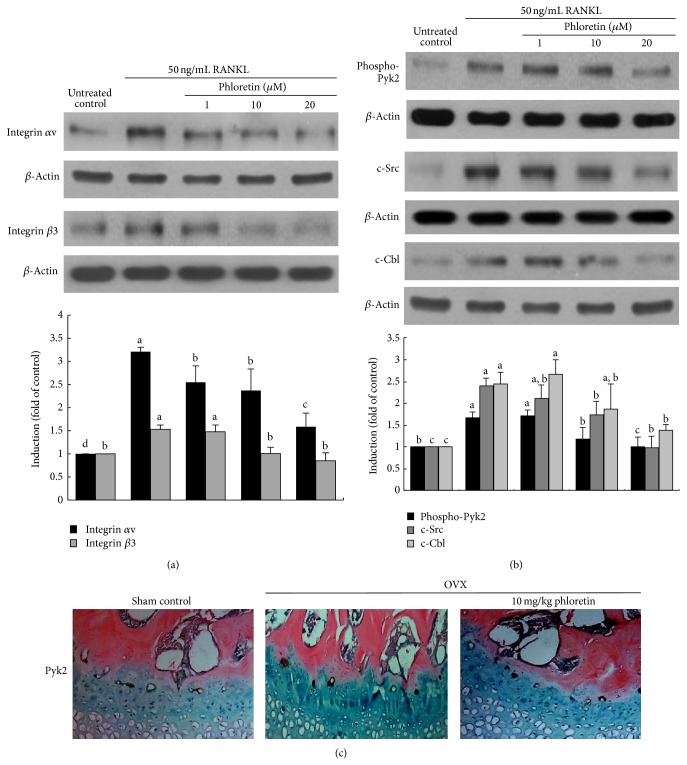
Blockade of RANKL-triggered integrin *α*v*β*3 induction (a) and c-Src-Pyk2-c-Cbl signaling (b) by phloretin, and inhibition of Pyk2 induction in ovariectomized (OVX) mouse femoral bone tissues (c). Raw 264.7 macrophages were cultured in *α*-MEM and exposed to 50 ng/mL RANKL for 5 days in the absence and presence of 1–20 *μ*M phloretin. OVX mice were orally treated with 10 mg/kg/day phloretin daily for 8 weeks. Cell lysates were subject to SDS-PAGE and Western blot analysis with a primary antibody against integrin *α*v, integrin *β*3, c-Src, phospho-Pyk2, and c-Cbl. Representative blot data were obtained from three independent experiments, and *β*-actin protein was used as an internal control. The bar graphs (mean ± SEM) in the bottom panels represent quantitative results of blots obtained from a densitometer. Respective values not sharing a common letter are different at *P* < 0.05. Immunohistochemical staining was conducted by using a primary antibody of mouse Pyk2 (brown spot staining) and by counterstaining with methyl green. Representative images were visualized under light microscopy (3 separate experiments). Magnification: 40-fold.

The adhesion receptor *α*v*β*3 integrin is involved in the maintenance of the sealing zone required for the effective osteoclastic bone resorption [15]. In this study the cellular induction of integrins of *α*v and *β*3 was elevated by the administration of 50 ng/mL RANKL to Raw 264.7 macrophages for 5 d (Figure 4(a)). In contrast, phloretin allayed such induction of both integrins of *α*v and *β*3.

In response to integrin *α*v*β*3 activation, Pyk2 is recruited to the adhesion structure podosomes through direct interactions with the *β*3 integrin cytoplasmic tails [28]. The nonreceptor tyrosine kinase Pyk2, a major cell adhesion-activated tyrosine kinase in osteoclasts, was activated by RANKL, which was suppressed by ≥10 *μ*M phloretin (Figure 4(b)). Another tyrosine kinase c-Src was induced in RANKL-stimulated Raw 264.7 cells, whereas ≥10 *μ*M phloretin dampened its induction (Figure 4(b)). Cbl is a key regulator of Src kinase activity and cell adhesion and migration [29]. Phloretin attenuated the induction of the adaptor protein in osteoclasts exposed to RANKL (Figure 4(b)). In femoral epiphyseal bone tissues of OVX mice the Pyk2 induction was elevated (Figure 4(c), brown spots). However, the oral administration of phloretin to OVX mice suppressed the Pyk2 induction (Figure 4(c)). Accordingly, phloretin may inhibit cytoskeletal organization and osteoclast function by disturbing formation of c-Src-Pyk2-Cbl complex in osteoclasts.

### 3.4. Effect of Phloretin on Paxillin Induction

**Figure 5:**
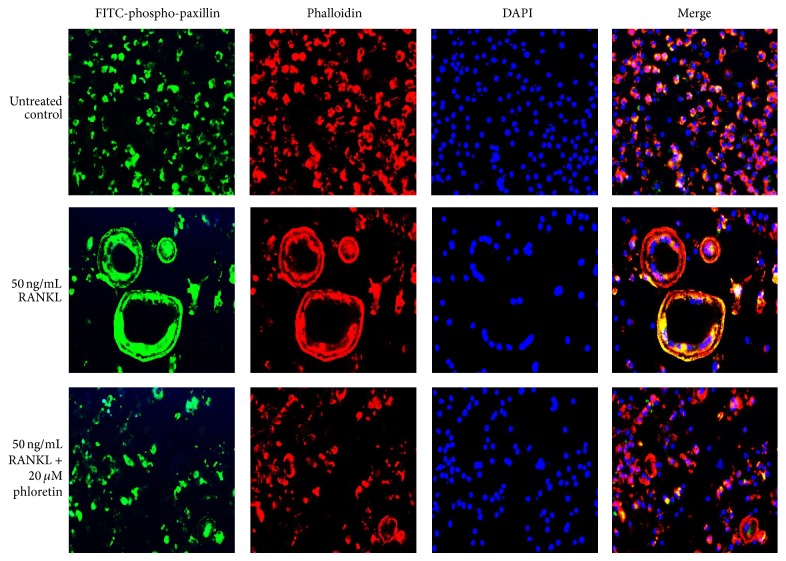
Inhibitory effects of phloretin on RANKL-induced paxillin activation and actin ring formation. Raw 264.7 macrophages were cultured in *α*-MEM for 5 days in the absence and presence of 1–20 *μ*M phloretin. RANKL-differentiated cells were fixed in 4% paraformaldehyde for 10 min. Immunocytochemical analysis was conducted with the primary phospho-paxillin antibody and anti-rabbit IgG conjugated with FITC. Subsequently, cells were incubated with phalloidin and counterstained with DAPI. Fluorescent images were taken with a fluorescence microscope. Original magnification of microscopic images (*n* = 3), 400-fold.

Paxillin is a multidomain adaptor localized at integrin-mediated focal adhesions and at the interface between the plasma membrane and the actin cytoskeleton [30]. Immunofluorescence staining showed that the distribution of phospho-paxillin (green) and F-actin (red) was very similar, with colocalization immediately inside peripheral podosome rings of RANKL-exposed osteoclasts (Figure 5). In contrast, the colocalization of F-actin to the periphery of the osteoclast is not observed in phloretin-experienced osteoclasts. The activated paxillin was absent in the cell periphery of phloretin-treated authentic osteoclasts (Figure 5).

**Figure 6:**
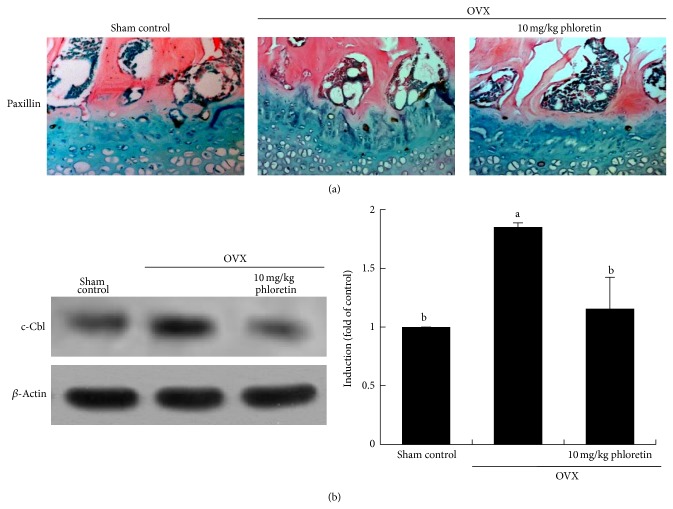
Inhibition of the induction of paxillin and c-Cbl in ovariectomized (OVX) mouse femoral bone tissues (a and b). OVX mice were orally treated with 10 mg/kg/day phloretin daily for 8 weeks. Immunohistochemical staining was conducted by using a primary antibody of mouse paxillin (brown spot staining) and by counterstaining with methyl green. Representative images were visualized under light microscopy (3 separate experiments). Magnification: 40-fold. Femoral bone tissue extracts were subject to SDS-PAGE and Western blot analysis with a primary antibody against mouse c-Cbl. Representative blot data were obtained from three independent experiments, and *β*-actin protein was used as an internal control. The bar graphs (mean ± SEM) in the right panels represent quantitative results of blots obtained from a densitometer. Respective values not sharing a common letter are different at *P* < 0.05.

This study further examined the induction of paxillin in epiphyseal bone tissues of estrogen-deficient animals. The paxillin protein was expressed in epiphyseal bone tissues of OVX mouse femora (Figure 6(a), brown spots). However, the oral administration of phloretin to OVX mice suppressed the paxillin induction. In addition, Western blot data revealed that the administration of phloretin to OVX mice suppressed the induction of c-Cbl in femoral bone tissues (Figure 6(b)).

### 3.5. Inhibition of Activation of PLC*γ* and Akt by Phloretin

**Figure 7:**
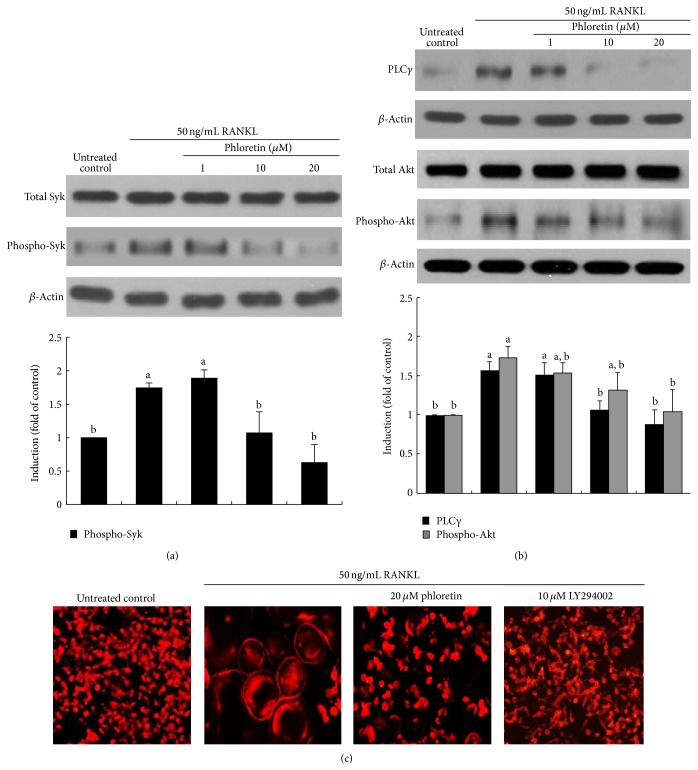
Inhibition of RANKL-induced Syk activation (a) and PLC*γ*-Akt signaling (b) by phloretin, and actin ring formation by PI3K inhibition (c). Raw 264.7 macrophages were cultured in *α*-MEM and exposed to 50 ng/mL RANKL for 5 days in the absence and presence of 1–20 *μ*M phloretin. Cell lysates were subject to SDS-PAGE and Western blot analysis with a primary antibody against Syk, phospho-Syk, PLC*γ*, Akt, and phospho-Akt. Representative blot data were obtained from three independent experiments, and *β*-actin protein was used as an internal control. The bar graphs (mean ± SEM) in the bottom panels represent quantitative results of blots obtained from a densitometer. Respective values not sharing a common letter are different at *P* < 0.05. Raw 264.7 cells were cultured for 5 days with 50 ng/mL RANKL in the presence of 20 *μ*M phloretin and 10 *μ*M LY294002 (PI3K inhibitor). Differentiated cells were fixed in 4% paraformaldehyde for 10 min and fluorescent rhodamine phalloidin was added to fixed cells. Fluorescent images were taken with a fluorescence microscope. Original magnification of microscopic images (*n* = 3), 400-fold.

The *α*v*β*3 integrin stimulates the resorptive capacity of the differentiated osteoclasts by organizing their cytoskeleton via the tyrosine kinase Syk [31]. As expected, this tyrosine kinase was highly activated in osteoclasts by RANKL (Figure 7(a)). In the current study phloretin allayed the induction of *α*v*β*3 integrin by RANKL (Figure 4(a)). Consistently, the RANKL-activated Syk was retarded in phloretin-treated osteoclasts. Thus, phloretin may block another kinase Syk-linked cytoskeleton organization in osteoclasts.

This study attempted to determine whether phloretin inhibited the PLC*γ* induction in RANKL-mediated osteoclasts. When phloretin was treated with mature osteoclasts exposed to RANKL, the induction of PLC*γ* was markedly attenuated (Figure 7(b)). The Akt activation was also deterred in 20 *μ*M phloretin-administrated osteoclasts. Accordingly, phloretin may morphologically disorganize actin cytoskeleton through blocking both Pyk2-paxillin-PLC*γ* signaling and Pyk2-paxillin-Akt signaling.

The phosphatidylinositol 3′-kinase- (PI3K-) Akt signaling pathway is activated by many types of cellular stimuli or toxic insults and regulates fundamental cellular functions [32]. When RANKL-exposed osteoclasts were treated with 20 *μ*M phloretin or 10 *μ*M LY294002, PI3K inhibitor, the actin ring formation was disrupted (Figure 7(c)). This result showed that the actin cytoskeleton organization entailed the PI3K-Akt signaling activation in mature osteoclasts.

## 4. Discussion

Estrogen deficiency after menopause frequently accelerates osteoclastic bone resorption, leading to postmenopausal osteoporosis characterized by osteopenia and fractures [6, 33]. Pharmacological therapies for postmenopausal osteoporosis include agents inhibiting bone resorption [34, 35]. However, the efficacy of phytoestrogens is not still definite [20]. Another investigation has shown that polyphenols such as hydroxytyrosol and EGCG prevent bone loss through blocking osteoclast formation and bone resorption [22, 23]. In our previous study phloretin reduced RANKL-stimulated resorptive activity in osteoclasts via retarding differentiation [24]. In addition, phloretin promoted osteoclast apoptosis and inhibited estrogen deficiency-induced osteoclastogenic resorption [25]. The current study further elucidated that phloretin manipulated protein kinase-signaling components responsible for the osteoclast cytoskeleton organization, which may display favorable effects in combating resorptive bone diseases.

Osteoclasts are multinucleated cells derived from hematopoietic lineages via a multifaceted differentiation process [2]. Excessive bone degradation by osteoclasts is associated with several osteolytic diseases such as postmenopausal osteoporosis or bone metastasis [3, 6]. The bone resorptive function of osteoclasts depends upon the integrity of the actin cytoskeleton and the formation of the actin-rich sealing zones [13, 14]. Upon adhesion of mature osteoclasts to bone, the osteoclasts alternate between stationary resorptive and migratory phases until dying of apoptosis [10]. The osteoclasts polarized on the bone-cell interface reorganize their cytoskeleton and membrane to form a unique domain with the sealing zone that is a distinct dense ring of F-actin-rich podosomes [13, 36]. In this study the RANKL-RANK contact by adding RANKL to macrophages highly induced the formation of F-actin-rich actin rings in osteoclasts, which was deterred by supplementing phloretin to RANKL-exposed macrophages. The formed sealing zones delimit the ruffled border, in which protons and proteases are released for the demineralization and degradation of the bone matrix [37]. The phloretin treatment retarded the RANKL induction of V-ATPase responsible for the bone demineralization [24]. Here phloretin lost dense ring-like pattern in osteoclasts and depleted the V-ATPase protein at the cell periphery.

The polarization process of osteoclasts occurs under the tutelage of the *α*v*β*3 integrin in collaboration with the receptor RANK [11, 15, 16]. During the osteoclast activation by RANKL, phloretin concomitantly inhibited the enhanced expression of *α*v and *β*3 integrins. Thus, phloretin may antagonize the osteoclast activation via demolishing normal cytoskeleton. Furthermore, RANKL induces c-Src to link to RANK in a *α*v*β*3-dependent fashion [16]. When occupied, *α*v*β*3 integrin activates a canonical signaling complex consisting of nonreceptor tyrosine kinases c-Src and proline-rich Pyk2 that permits the cell to form actin rings [13, 29]. This study found that RANKL stimulated the induction of c-Src and the phosphorylation of Pyk2 and Syk, which was blunted by phloretin. Since phloretin inhibited the induction of c-Src and kinases pivotal in organizing the cell's cytoskeleton, one can assume that this compound can encumber reorganization of focal adhesions and cell migration. In femoral bone tissues of OVX mice with decreased trabecular collagen formation, the epiphyseal induction of Pyk2 was observed.

Pyk2 and Syk are structurally recruited to a signaling complex downstream to the activation of paxillin and PLC*γ* [30, 31]. It can be speculated that the activation of Pyk2-PLC*γ* signaling results in the Akt activation responsible for the actin cytoskeleton reorganization. In the present study paxillin was colocalized at the periphery of the osteoclasts in distribution with F-actin crucial to cytoskeleton formation. In addition to the inhibition of Pyk2 activation by phloretin, it demoted the induction and activation of paxillin in mature osteoclasts and in femoral bone tissues of OVX mice. Additionally, phloretin deterred PLC*γ* and Akt from being induced and activated in RANKL-treated osteoclasts, indicating that this compound may prevent the reorganization of focal adhesions and cell migration for the actin cytoskeleton formation entailing c-Src-Pyk2/Syk-paxillin-PLC*γ*-Akt signaling.

Absence of any of the signaling molecules in the *α*v*β*3-activated sequence compromises osteoclast cytoskeletal organization and abridges bone resorption [15, 16]. Here phloretin appeared to allay estrogen deficiency-induced bone resorption through blocking the c-Src downstream signaling of Pyk2 and paxillin. This study attempted to explore the precise inhibitory mechanism(s) by which phloretin abrogated actin cytoskeleton reorganization pertaining to bone resorption in postmenopausal osteoporosis. Phloretin inhibited the degradation of bone matrix occurring in sealed lacunae beneath the ruffled border of osteoclasts [24]. In spite of the V-ATPase inhibition by phloretin at the periphery of osteoclasts, it did not influence the induction of gelsolin and vinculin in RANKL-exposed cells. Instead, phloretin prevented the activation of the actin-binding protein vinculin. The actin-binding proteins are involved in the linkage of integrin adhesion molecules to the actin cytoskeleton [38, 39]. Oral administration of phloretin in OVX mice may disorganize cytoskeleton of osteoclastic cells through obviating the induction of gelsolin and vinculin. However, there are other bone tissue specific mechanisms of phloretin to be further considered. Since glucose transporter-1 is expressed in osteoclasts [40], phloretin may inhibit glucose transporter-1 induction during osteoclast activation in postmenopausal osteoporosis.

In summary, the current report demonstrated that phloretin abrogated *α*v*β*3 integrin-c-Src-mediated actin cytoskeletal reorganization responsible for bone resorption. This compound inhibited RANKL-mediated actin ring formation through disturbing the sequential molecular events of Pyk2 and Syk. The oral administration of phloretin allayed the Pyk2-paxillin signaling pathway leading to the induction of the actin-binding proteins in femoral bone tissues of OVX mice with decreased trabecular ECM. Therefore, phloretin may be effective in ameliorating pathological osteoresorptive disorders such as postmenopausal osteoporosis.

## References

[1] Sheng M. H. C., Lau K. H. W. (2009). Role of protein-tyrosine phosphatases in regulation of osteoclastic activity. *Cellular and Molecular Life Sciences*.

[2] Bar-Shavit Z. (2007). The osteoclast: a multinucleated, hematopoietic-origin, bone-resorbing osteoimmune cell. *Journal of Cellular Biochemistry*.

[3] Rachner T. D., Khosla S., Hofbauer L. C. (2011). Osteoporosis: now and the future. *The Lancet*.

[4] Lane N. E., Yao W., Nakamura M. C. (2005). Mice lacking the integrin *β*5 subunit have accelerated osteoclast maturation and increased activity in the estrogen-deficient state. *Journal of Bone and Mineral Research*.

[5] Hofbauer L. C., Heufelder A. E. (2001). Role of receptor activator of nuclear factor-*κ*B ligand and osteoprotegerin in bone cell biology. *Journal of Molecular Medicine*.

[6] Faienza M. F., Ventura A., Marzano F., Cavallo L. (2013). Postmenopausal osteoporosis: the role of immune system cells. *Clinical and Developmental Immunology*.

[7] Kim J.-L., Kim Y.-H., Kang M.-K., Gong J.-H., Han S.-J., Kang Y.-H. (2013). Antiosteoclastic activity of milk thistle extract after ovariectomy to suppress estrogen deficiency-induced osteoporosis. *BioMed Research International*.

[8] Chen J. S., Sambrook P. N. (2011). Antiresorptive therapies for osteoporosis: a clinical overview. *Nature Reviews Endocrinology*.

[9] Åkesson K. (2003). New approaches to pharmacological treatment of osteoporosis. *Bulletin of the World Health Organization*.

[10] Touaitahuata H., Blangy A., Vives V. (2014). Modulation of osteoclast differentiation and bone resorption by Rho GTPases. *Small GTPases*.

[11] Novack D. V., Faccio R. (2011). Osteoclast motility: putting the brakes on bone resorption. *Ageing Research Reviews*.

[12] Coury F., Zenger S., Stewart A. K. (2013). SLC4A2-mediated Cl^−^/HCO_3_
^−^ exchange activity is essential for calpain-dependent regulation of the actin cytoskeleton in osteoclasts. *Proceedings of the National Academy of Sciences of the United States of America*.

[13] Teitelbaum S. L. (2011). The osteoclast and its unique cytoskeleton. *Annals of the New York Academy of Sciences*.

[14] Jurdic P., Saltel F., Chabadel A., Destaing O. (2006). Podosome and sealing zone: specificity of the osteoclast model. *European Journal of Cell Biology*.

[15] Nakamura I., Duong L. T., Rodan S. B., Rodan G. A. (2007). Involvement of *α*v*β*3 integrins in osteoclast function. *Journal of Bone and Mineral Metabolism*.

[16] Izawa T., Zou W., Chappel J. C., Ashley J. W., Feng X., Teitelbaum S. L. (2012). c-Src links a RANK/*α*v*β*3 integrin complex to the osteoclast cytoskeleton. *Molecular and Cellular Biology*.

[17] Al-Anazi A. F., Qureshi V. F., Javaid K., Qureshi S. (2011). Preventive effects of phytoestrogens against postmenopausal osteoporosis as compared to the available therapeutic choices: an overview. *Journal of Natural Science, Biology and Medicine*.

[18] Banu J., Varela E., Fernandes G. (2012). Alternative therapies for the prevention and treatment of osteoporosis. *Nutrition Reviews*.

[19] Turner J. V., Agatonovic-Kustrin S., Glass B. D. (2007). Molecular aspects of phytoestrogen selective binding at estrogen receptors. *Journal of Pharmaceutical Sciences*.

[20] Bedell S., Nachtigall M., Naftolin F. (2014). The pros and cons of plant estrogens for menopause. *Journal of Steroid Biochemistry and Molecular Biology*.

[21] Taku K., Melby M. K., Kronenberg F., Kurzer M. S., Messina M. (2012). Extracted or synthesized soybean isoflavones reduce menopausal hot flash frequency and severity: systematic review and meta-analysis of randomized controlled trials. *Menopause*.

[22] Hagiwara K., Goto T., Araki M., Miyazaki H., Hagiwara H. (2011). Olive polyphenol hydroxytyrosol prevents bone loss. *European Journal of Pharmacology*.

[23] Kamon M., Zhao R., Sakamoto K. (2010). Green tea polyphenol (-)-epigallocatechin gallate suppressed the differentiation of murine osteoblastic MC3T3-E1 cells. *Cell Biology International*.

[24] Kim J.-L., Kang M.-K., Gong J.-H., Park S.-H., Han S.-Y., Kang Y.-H. (2012). Novel antiosteoclastogenic activity of phloretin antagonizing RANKL-induced osteoclast differentiation of murine macrophages. *Molecular Nutrition and Food Research*.

[25] Lee E. J., Kim J. L., Kim Y. H., Kang M. K., Gong J. H., Kang Y. H. (2014). Phloretin promotes osteoclast apoptosis in murine macrophages and inhibits estrogen deficiency-induced osteoporosis in mice. *Phytomedicine*.

[26] Hara K., Kobayashi M., Akiyama Y. (2007). Influence of bone osteocalcin levels on bone loss induced by ovariectomy in rats. *Journal of Bone and Mineral Metabolism*.

[27] Qin A., Cheng T. S., Pavlos N. J., Lin Z., Dai K. R., Zheng M. H. (2012). V-ATPases in osteoclasts: structure, function and potential inhibitors of bone resorption. *International Journal of Biochemistry and Cell Biology*.

[28] Xiong W.-C., Feng X. (2003). PYK2 and FAK in osteoclasts. *Frontiers in Bioscience*.

[29] Sanjay A., Houghton A., Neff L. (2001). Cbl associates with Pyk2 and Src to regulate Src kinase activity, *α*
_v_
*β*
_3_ integrin-mediated signaling, cell adhesion, and osteoclast motility. *Journal of Cell Biology*.

[30] Turner C. E. (2000). Paxillin and focal adhesion signalling. *Nature Cell Biology*.

[31] Zou W., Croke M., Fukunaga T., Broekelmann T. J., Mecham R. P., Teitelbaum S. L. (2013). Zap70 inhibits Syk-mediated osteoclast function. *Journal of Cellular Biochemistry*.

[32] Lee Z. H., Kim H.-H. (2003). Signal transduction by receptor activator of nuclear factor kappa B in osteoclasts. *Biochemical and Biophysical Research Communications*.

[33] Stein E. M., Kepley A., Walker M. (2014). Skeletal structure in postmenopausal women with osteopenia and fractures is characterized by abnormal trabecular plates and cortical thinning. *Journal of Bone and Mineral Research*.

[34] Beard M. K. (2012). Bisphosphonate therapy for osteoporosis: combining optimal fracture risk reduction with patient preference. *Current Medical Research and Opinion*.

[35] Dawson-Hughes B., Bischoff-Ferrari H. A. (2007). Therapy of osteoporosis with calcium and vitamin D. *Journal of Bone and Mineral Research*.

[36] Takahashi N., Ejiri S., Yanagisawa S., Ozawa H. (2007). Regulation of osteoclast polarization. *Odontology*.

[37] Szewczyk K. A., Fuller K., Chambers T. J. (2013). Distinctive subdomains in the resorbing surface of osteoclasts. *PLoS ONE*.

[38] Beaulieu V., Da Silva N., Pastor-Soler N. (2005). Modulation of the actin cytoskeleton via gelsolin regulates vacuolar H^+^-ATPase recycling. *The Journal of Biological Chemistry*.

[39] Fukunaga T., Zou W., Warren J. T., Teitelbaum S. L. (2014). Vinculin regulates osteoclast function. *Journal of Biological Chemistry*.

[40] Knowles H. J., Athanasou N. A. (2008). Hypoxia-inducible factor is expressed in giant cell tumour of bone and mediates paracrine effects of hypoxia on monocyte-osteoclast differentiation via induction of VEGF. *Journal of Pathology*.

